# Composition of the rumen archaeal community of growing camels fed different concentrate levels

**DOI:** 10.1007/s10123-023-00459-6

**Published:** 2023-12-07

**Authors:** Alaa Emara Rabee, Ahmed R. Askar

**Affiliations:** Animal and Poultry Nutrition Department, Desert Research Center, Ministry of Agriculture and Land Reclamation, Cairo, Egypt

**Keywords:** Camel, Feeding, Concentrate level, Rumen, Archaea

## Abstract

Understanding the rumen fermentation and methanogenic community in camels fed intensively is critical for optimizing rumen fermentation, improving feed efficiency, and lowering methane emissions. Using Illumina MiSeq sequencing, quantitative real-time PCR, and high-performance liquid chromatography, this study evaluates the influence of different concentrate supplement levels in the diet on rumen fermentation as well as the diversity and structure of the rumen methanogenic community for growing dromedary camels. Twelve growing camels were divided into three groups and given three levels of concentrate supplement, 0.7% (C1), 1% (C2), and 1.3% (C3) based on their body weight. All animals were fed alfalfa hay ad libitum. The levels of total volatile fatty acid, rumen ammonia, and methanogen copy number were unaffected by the supplementation level. Increasing the concentrate supplement level increased the proportion of propionic acid while decreasing the proportion of acetic acid. Increasing the level of concentrate in the diet had no effect on alpha diversity metrics or beta diversity of rumen methanogens. *Methanobrevibacter* and *Methanosphaera* predominated the methanogenic community and were declined as concentrate supplement level increased. This study sheds new light on the effect of concentrate supplement level in growing camels’ diet on rumen fermentation and methanogenic community, which could help in the development of a strategy that aimed to reduce methane emissions and enhance feed efficiency.

## Introduction

Rumen methanogens are the primary producers of methane (CH_4_) in the rumen. They restrict fermentation gases from accumulation in the rumen by utilizing hydrogen (H_2_) to convert carbon dioxide to CH_4_ via methanogenesis (Hook et al., 2010). Methanogens also use format and other fermentation byproducts as an energy source to create methane. Methanogenesis keeps the rumen’s hydrogen pressure low, which promotes anaerobic fermentation of ingested feed (Carberry et al. 2014a). Methane, on the other hand, represents a 2–12% loss in dietary gross energy intake and increases greenhouse gas emissions (Rabee et al. 2020).

Rumen fermentation is carried out through the interaction between bacteria, protozoa, fungi, and methanogenic archaea. These microorganisms collaborate to produce volatile fatty acids, ammonia (NH_3_-N), H_2_, and CO_2_ from the ingested feed (Zhang et al. 2017). Understanding the factors that influence rumen fermentation and methane generation in different ruminant species is critical for developing an efficient approach for reducing methane emissions and improving feed efficiency (Tseten et al. 2022). Dietary modulation is the primary technique for influencing rumen microbial fermentation and CH_4_ generation (Haque 2018). The ratio of concentrates to roughage and starch content are major dietary factors that influence rumen fermentation and methane generation. Previous research (Popova et al. 2011; Doreau et al. 2011) showed that increasing the starch component of cow diets reduced CH_4_ generation. Zhang et al. (2017), on the other hand, investigated the effect of forage-to-concentrate ratios on rumen methanogen changes in Holstein heifers and discovered that the methanogenic community was dominated by the genus *Methanobrevibacter* and the relative abundances of rumen methanogenic genera were not significantly affected by dietary concentrate levels. Rabee et al. (2022) showed a small decrease in the relative abundance of the genus *Methanobrevibacter* in sheep diets as a result of the substitution of concentrate feed combination with date palm byproducts and olive cake. According to Vaidya et al. (2020), silage type (grass or maize silage) was positively linked with the abundance of the species *Methanobrevibacter* and *Methanosphaera*. Understanding the modulations of methanogenic communities caused by diet alterations in various animal species, including camels, could thus accelerate efforts to reduce methane emissions from the livestock sector (Dittmann et al. 2014; Guerouali and Laabouri 2018; Haque 2018).

In light of climate change, the significance of camels in attaining food and economic security becomes apparent (Kandil et al. 2023). They have excellent adaptive properties for drought, are well adapted to arid environments, desertification, and scarce natural resources, and are a major source of income for people living in arid and semi-arid regions (Askar 2019), contributing to the sustainable development of desert regions (Gagaoua et al. 2022; Kandil et al. 2023) and being the most productive livestock species for milk and meat under these harsh conditions (Seifu 2023). As the demand for camel milk and meat increases, numerous efforts have been made to intensify camel production (Kandil et al. 2023). However, the scientific community paid less attention to camels than to other livestock species. Earlier research has shown that camels emit less methane than cattle and livestock (Dittmann et al. 2014; Guerouali and Laabouri 2018). Consequently, it is essential to understand camel metabolism and rumen microbial fermentation. Recently, the rumen microbiome of camels has received more attention than in the past; however, compared to other domesticated ruminants, the rumen microbiome of camels remains less investigated. Furthermore, most rumen microbial community studies, including rumen methanogens, are surveys (Rabee et al. 2020; Askar et al. 2023). Rabee et al. (2020) used cDNA sequencing to investigate the metabolically active archaea in camel rumen under various feeding systems and reported that rumen methanogens were classified into the order Thermoplasmatales and the genera *Methanobrevibacter*, *Methanomicrobium*, *Methanosphaera*, and *Methanobacterium*. Askar et al. (2023) reported the effect of concentrate supplement levels on the rumen bacterial community in growing camels. In addition, the effect of concentrate level and diet composition on rumen methanogens in other ruminants has been well-documented (Zhang et al. 2017; Vaidya et al. 2020; Rabee et al. 2022). However, the effect of concentrate level in the diet on the community of rumen methanogens in growing camels has not yet been reported. Thus, the purpose of this study was to investigate the effect of three levels of concentrate supplement on rumen fermentation and the diversity and composition of rumen methanogens in growing camels.

## Material and methods

The project was approved, and all samples were collected according to the guidelines of the Animal Care and Use Committee, Desert Research Center, Egypt. All methods used in the experiment were performed according to the ARRIVE guidelines. Furthermore, the study does not include euthanasia of the experimental camels. The project was approved by the Institutional Animal Care and Use Committee, Faculty of Veterinary Medicine, University of Sadat City, Egypt (Reference: VUSC00008), and the experiment does not include animal euthanasia.

### Animals and treatments

The experiment was carried out at Ras-Sudr Research Station that belongs to Desert Research Center, South Sinai Governorate, Egypt. However, the experimental procedures, including animals, treatments, and experimental diets, were previously described in Askar et al. (2023). Fifteen growing male camels (*Camelus dromedarius*, 12 months old, 305 ± 8.75 kg body weight) were divided into three groups, each with five camels, and given one of the three concentrate supplement levels based on their body weight (BW), 0.7 (C1), 1.0 (C2), and 1.3% (C3) of BW. Camels received free access to drinking water and *ad-lib* alfalfa hay as sole roughage. The experiment lasted 6 months, and the amount of feed refusal was recorded on a regular basis. The concentrate feed mixture consisted of 55% corn, 15% soybean meal, 10% cottonseed meal, 15% wheat bran, 2.5% limestone, 1.5% salt, 0.5% sodium bicarbonate, 0.3% premix, 0.1% yeast, and 0.1% antitoxins. The proximate chemical composition for alfalfa hay was 938 g/kg DM, 809 g/kg OM, 141 g/kg CP, and 464 g/kg NDF. Moreover, the proximate chemical composition for the concentrate feed mixture was 946 g/kg DM, 874 g/kg OM, 156 g/kg CP, and 342 g/kg NDF.

### Rumen samples

At the end of the experimental period, rumen samples were collected 3 h after the morning feeding using a stomach tube and strained through cheesecloth layers. Rumen PH was recorded using a digital pH meter (WPA CD70). Rumen samples, then, were used to estimate rumen ammonia, volatile fatty acids (VFAs), and isolated rumen DNA.

### Analytical procedures

The dietary dry matter (DM), acid detergent fiber (ADF), neutral detergent fiber (NDF), and crude protein (CP) were determined in the alfalfa hay and concentrate mixture. DM was determined by drying at 105 °C for 24 h. The neutral detergent fiber (NDF) and acid detergent fiber (ADF) contents were determined according to Mertens (2002), and AOAC (2005). Moreover, CP was determined according to AOAC (2005). The rumen ammonia and total VFA concentrations were determined by steam distillation in Kjeldahl distillation equipment according to the methods of Kholif et al. (2023) and AOAC (2005). In addition, individual VFAs were measured using high-performance liquid chromatography (HPLC) using C18 column and % phosphoric acid as a mobile phase.

### DNA extraction, PCR amplification, and sequencing

One milliliter of rumen sample was centrifuged at 13,000 rpm for 15 min, and the remained pellets were used for DNA extraction using i-genomic Stool DNA Extraction Mini Kit (iNtRON Biotechnology, Inc.) according to the manufacturer’s instructions. Then, DNA quantity and quality were checked by agarose gel and Nanodrop spectrophotometer. Archaeal 16S rDNA gene was amplified using primers Ar915aF (5-AGGAATTGGCGGGGGAGCAC-3) and Ar1386R (5-GCGGTGTGTGCAAGGAGC-3) (Rabee et al. 2022). The PCR conditions were as follows: 95 °C for 5 min; 30 cycles 95 °C for 20 s, 55 °C for 15 s, 72 °C for 5 min, and 72 °C for 10 min. The PCR amplicons were purified and sequenced using Illumina MiSeq sequencing.

### Determination of copy number of archaeal 16S rRNA by using Quantitative PCR (qPCR)

The qPCR was carried out to measure the total copy number of archaeal 16S rDNA in 1 µL of isolated DNA. Standards were generated using serial dilutions of purified DNA from *Methanobrevibacter ruminantium*, and *Methanosphaera stadtmanae* purchased from Deutsche Sammlung von Mikroorganismen und Zellkulturen (DSMZ), Braunschweig, Germany. The standard curve was created using a dilution series of the standards ranging from 10^1^ to 10^6^ copies of the 16S rDNA. The qPCR was performed using the Applied Biosystems StepOne system (Applied Biosystems, Foster City, USA). The archaeal-specific primers Arch 1174–1195 F (5-GAGGAAGGAGTGGACGACGGTA-3) and Arch 1406–1389 R (5-ACGGGCGGTGTGTGCAAG-3) (Rabee et al. 2022) were used to amplify DNA samples and diluted standards. The 10-µL qPCR reaction contained 1µL genomic DNA, 1 μL of each primer, and 7 μL of SYBER Green qPCR- master mix (iNtRON Biotechnology, Inc.). The qPCR conditions were 40 cycles of 95 °C for 15 s, and 60 °C for 60 s. The total copy number of archaeal 16S rDNA per 1 µL of DNA was determined relying on the linear relationship between the threshold amplification (Ct) and the logarithm of 16S rDNA copy numbers of the standards.

### Bioinformatics analysis

All the paired-end (PE) Illumina raw sequences were processed in R (version 3.5.2) using the DADA2 (version 1.11.3) pipeline as described by Callahan et al. (2016). First, quality checks were conducted; clean reads were denoised, dereplicated, and filtered for chimeras to generate Amplicon Sequence Variants (ASVs). The taxonomic assignment of sequence variants was performed using a combination of the functions assign Taxonomy and assignSpecies and was compared using the SILVA reference database. Various alpha diversity indices, Chao1, Shannon, and InvSimpson were obtained. Beta diversity was assessed as the principal coordinate analysis (PCoA) based on bray–curtis dissimilarity.

### Statistical analysis

The statistical analyses were conducted using the IBM SPSS version 20 software (IBM Corp. 2011). The differences in feed intake, rumen fermentation parameters, archaeal copy number, archaeal diversity, and relative abundance of archaeal genera were performed using one-way ANOVA using the Tukey test. Differences with *P* < 0.05 were accepted as statistically significant.

## Results

### Feed intake and rumen fermentation

**Fig. 1:**
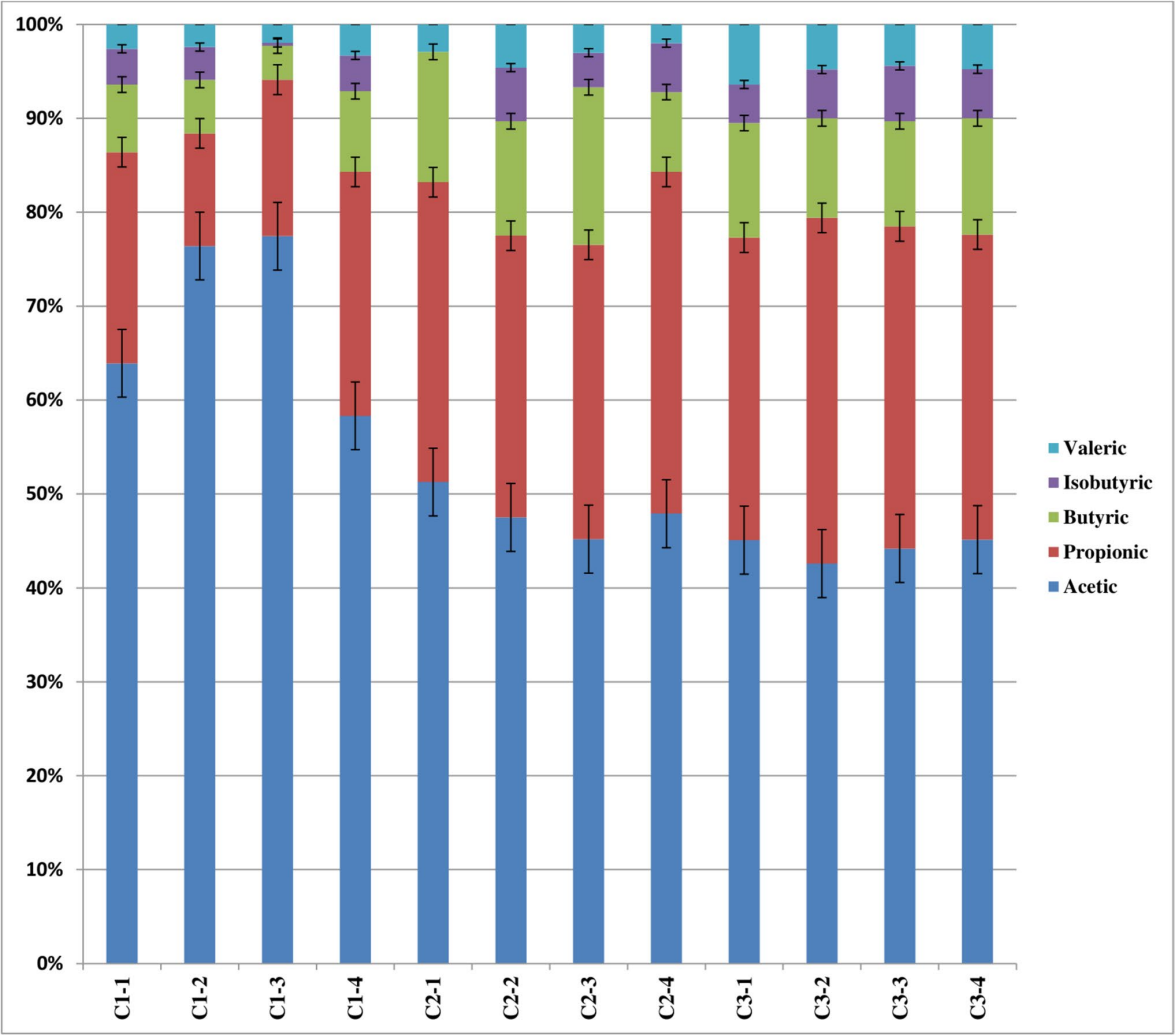
The percentages of rumen volatile fatty acids for growing camels as affected by feeding different concentrate supplement levels in the diet

The mean values of feed intake expressed as g/kg metabolic body weight (kg BW^0.75^) and rumen fermentation parameters are shown in Table 1 and Fig. 1. The results demonstrated that increasing the level of concentrate supplement in the diet increased the amount of dry matter intake, including that of crude protein and fiber. However, rumen ammonia, total VFA, and total methanogens copy number were not affected by increasing the concentrate supplement level (Table 1). In addition, camels fed a low level of concentrate level had a greater proportion of acetic acid (C1, 69.4%, *P* < 0.05) than those fed a medium (C2, 48.7%) or high level of concentrate (C3, 44.8%) (Fig. 1). Increasing the level of concentrate supplement in the diet markedly (*P* < 0.05) increased the proportion of propionic acid (C3, 34.4%), followed by C2 (32.9%) and C1 (19.9%). Moreover, camels fed a medium level of concentrate supplement (C2) had a significantly (*P* < 0.05) greater proportion of butyric acid (13.1%), followed by those fed C3 (11.8%) and C1 (6.3%), respectively (Fig. 1).

**Table 1:** Feed intake, rumen fermentation parameters, and methanogens copy number for growing camels as affected by dietary concentrate levels

Items	Concentrate supplement level in diet						Mean	SEM	*P* value
	Low (C1)		Medium (C2)		High (C3)				
	Mean	SE	Mean	SE	Mean	SE			
Feed intake, g/kg BW^0.75^									
Dry matter	67.1	1.24	60.9	2.06	72.5	1.60	66.9	1.55	0.001
Crude protein	9.9	0.17	9.1	0.28	10.9	0.22	9.9	0.22	0.001
Neutral detergent fiber	27.3	0.60	24.0	1.07	28.4	0.79	26.6	0.671	0.009
Rumen fermentation parameters									
Ammonia	47.6	3.40	36.9	4.44	43.9	6.97	42.8	3.01	0.37
Total VFAs	28.0	1.35	25.2	1.58	23.5	2.77	25.6	1.186	0.32
Methanogens population*	3.2	0.16	3.5	0.04	3.0	0.14	3.2	0.09	0.06

^*^Log_10_ copies/µL DNA; *SEM* stander error of means, *Total VFAs* total volatile fatty acids

### Diversity of rumen archaea

**Fig. 2:**
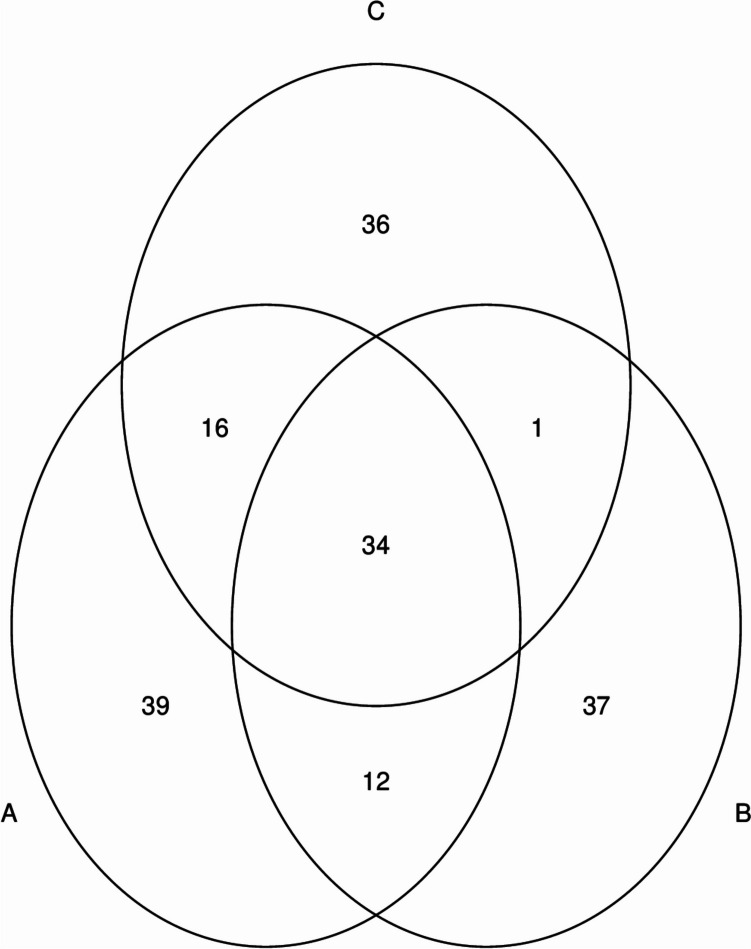
Venn diagram shows the number of archaeal taxa shared between camel groups C1 (A), C2 (B), and C3 (C). Each circle represents a camel group and the overlapping areas represent the common archaeal taxa

**Fig. 3:**
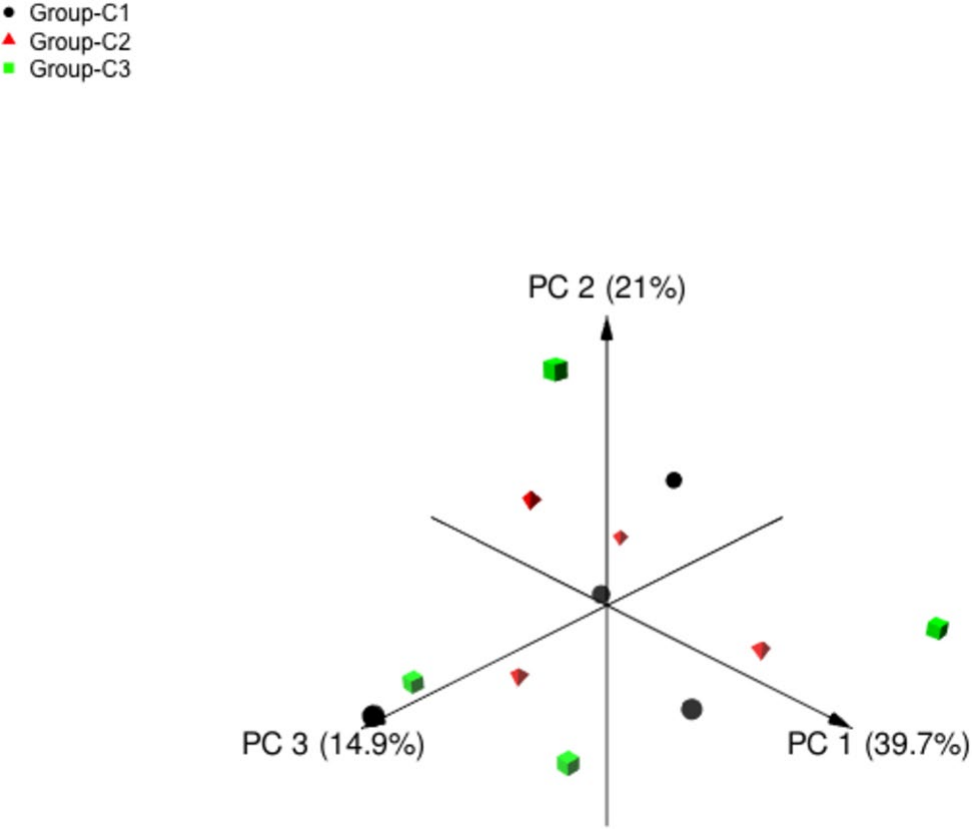
Principal coordinates analysis (PCoA) of rumen archaeal community in camels received different concentrate levels. Black circles for C1 group, red triangles for C2 group, and green squares for C3 group

The sequencing of the V4 region on the archaeal 16S rDNA gene in 12 (*n* = 4) rumen samples resulted in 284,801 high-quality sequence reads with an average of 23,733.42 (Table 2). The mean number of observed ASVs was 75.91 ± 5.83 and camel group C3 had higher ASVs (87.5 ± 6.02) followed by C2 (72.25 ± 7.12), and C1 (68 ± 14.738); however, the difference was not significant. Venn diagram showed that 34 archaeal ASVs were shared between the three camel groups (Fig. 2). Beta diversity of camel rumen archaeal community was calculated and viewed using principal coordinate analysis based (PCoA) on the Bray–Curtis distances (Fig. 3). The result showed no clear clustering of camel rumen samples based on the dietary concentrate level (Fig. 3). Increasing the dietary concentrate level did not impact the number of ASVs and alpha diversity indices, Chao1, Shannon, Simpson, and InvSimpson, significantly. However, camel group C3 that received a higher concentrate level showed higher archaeal diversity (Table 2).

**Table 2:** Diversity of the methanogens community in the rumen of growing camels supplemented with different concentrate levels

	Low (C1)		Medium (C2)		High (C3)		Mean	SE	*P* value
	Mean	SE	Mean	SE	Mean	SE			
Sequence reads	21,889	5600.33	34,842	18,859.96	14,469.25	712.44	23,733.42	6455.76	0.47
Observed ASVs	68	14.738	72.25	7.12	87.5	6.02	75.91	5.83	0.39
Chao1	68	14.73	72.5	6.89	87.65	6.01	76.05	5.81	0.38
Shannon	2.47	0.26	2.05	0.42	2.61	0.24	2.38	0.18	0.46
Simpson	0.82	0.047	0.68	0.13	0.81	0.06	0.77	0.050	0.52
InvSimpson	6.90	1.86	4.79	1.29	7.95	2.82	6.55	1.16	0.57

### Structure of camel rumen archaea

All the archaeal community in the camel rumen was assigned to the phylum Euryarchaeota. The members of this phylum were further classified into four orders Methanobacteriales, Methanosarcinales, Methanomassiliicoccales, and Methanomicrobiales. The members of the order Methanobacteriales were assigned to the family Methanobacteriaceae, which was further classified into genus *Methanobrevibacter*, *Methanosphaera*, and *Methanobacterium* (Table 3). Genus *Methanobrevibacter* dominated the archaeal community and represented 96.18% of the methanogenes community. This genus showed a significant decrease (*P* < 0.05) by increasing the concentrate level. A similar trend was observed with the genus *Methanosphaera*, which represented 1.18% of the methanogenic community. In contrast, the relative abundance of genus *Methanobacterium* was increased significantly (*P* < 0.05) by increasing the concentrate level.

**Table 3:** Relative abundance (%) of archaeal genera in the rumen of growing camels supplemented with different concentrate levels

	Low (C1)		Medium (C2)		High (C3)		Mean	SE	*P* value
	Mean	SE	Mean	SE	Mean	SE			
Methanobrevibacter	97.62	0.40	96.59	0.36	94.32	0.53	96.18	0.47	0.001
Methanosphaera	1.60	0.10	1.26	0.44	0.68	0.15	1.18	0.18	0.11
Methanobacterium	0.15	0.06	0.57	0.19	1.44	0.39	0.72	0.21	0.01
Methermicoccus	0.44	0.06	0.70	0.23	1.35	0.37	0.83	0.17	0.08
Methanosarcina	0.45	0.05	0.92	0.25	1.88	0.48	1.09	0.24	0.03
Methanomassiliicoccus	0.14	0.03	0.28	0.14	0	0	0	0	0
Methanocorpusculum	0.03	0.03	0.07	0.03	0.14	0.07	0.08	0.02	0.25
Methanomicrobiales_Unclassified	0.02	0.02	0.035	0.0037	0	0	0	0	0

The member of order Methanosarcinales was classified into two families, Methermicoccaceae that was assigned to genus *Methermicoccus*, and family Methanosarcinaceae, which was further classified into genus *Methanosarcina*. This genus was increased by increasing the concentrate level (*P* < 0.05) (Table 3). Moreover, order Methanomassiliicoccales was assigned to family Methanomassiliicoccaceae and genus *Methanomassiliicoccus* that was not detected in group C3 (Table 3). In addition, order Methanomicrobiales was further classified into family Methanocorpusculaceae and unclassified family that was not detected in C3. Family Methanocorpusculaceae was assigned to genus *Methanocorpusculum* that was increased by increasing the concentrate level (Table 3).

### Principal component analysis (PCA)

**Fig. 4:**
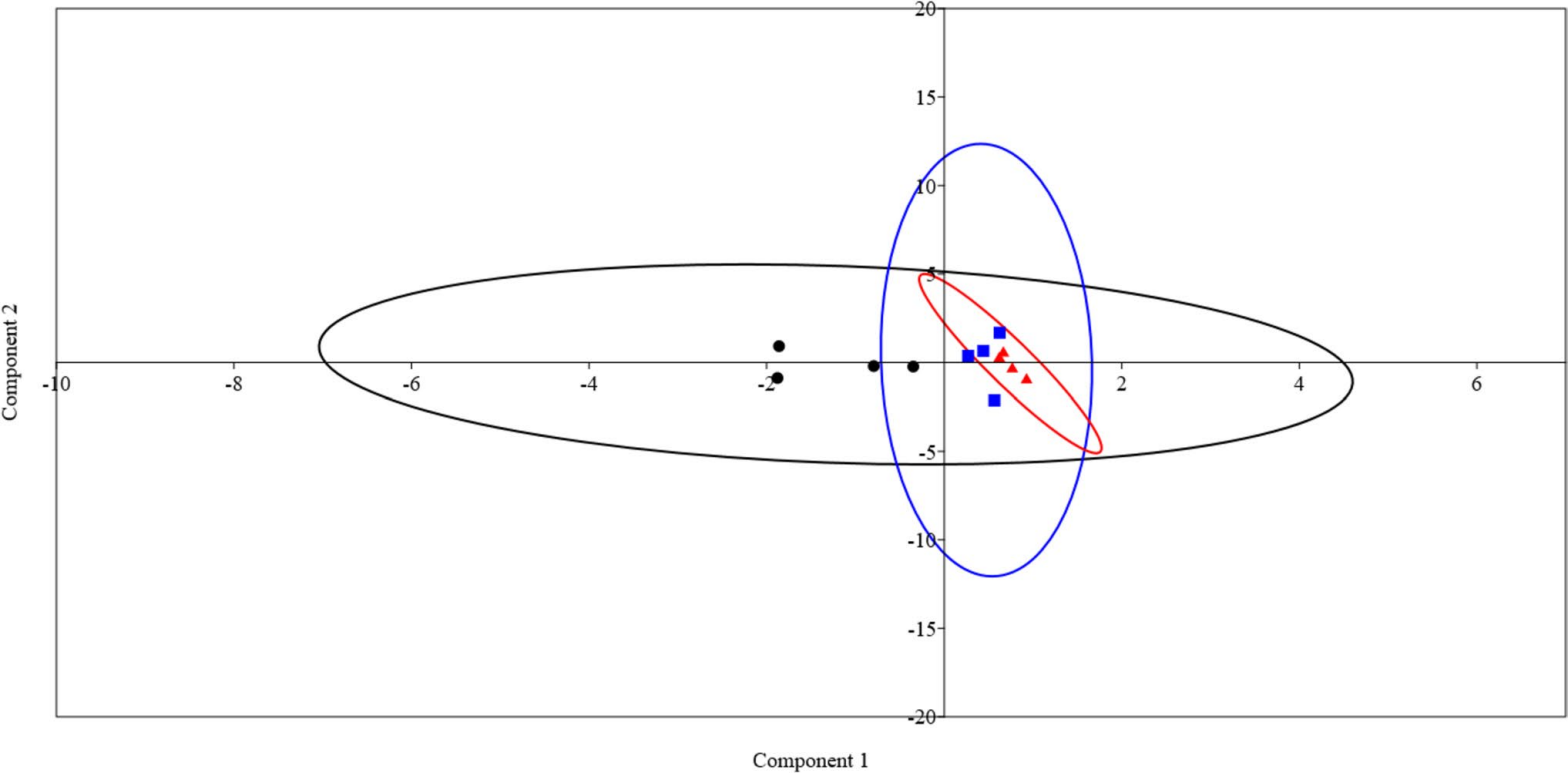
Principal component analysis (PCA) based on concentrations of volatile fatty acids and the relative abundances of rumen methanogens in the rumen of camel groups received different concentrate levels; black circles for C1 group, blue squares for C2 group, and red triangles for C3 group

PCA analysis was conducted based on the concentration of VFAs, acetic, propionic, butyric, isobutyric, and valeric; and relative abundances of archaeal genera, *Methanobrevibacter*, *Methermicoccus*, *Methanosarcina*, *Methanosphaera*, *Methanobacterium*, and *Methanocorpusculum*. The results showed that the rumen samples were separated distinctly based on animal diet (Fig. 4). The differences between groups were driven by the VFAs and relative abundance of genus *Methanobrevibacter*.

### Correlation relationships between rumen methanogens and rumen fermentation

**Fig. 5:**
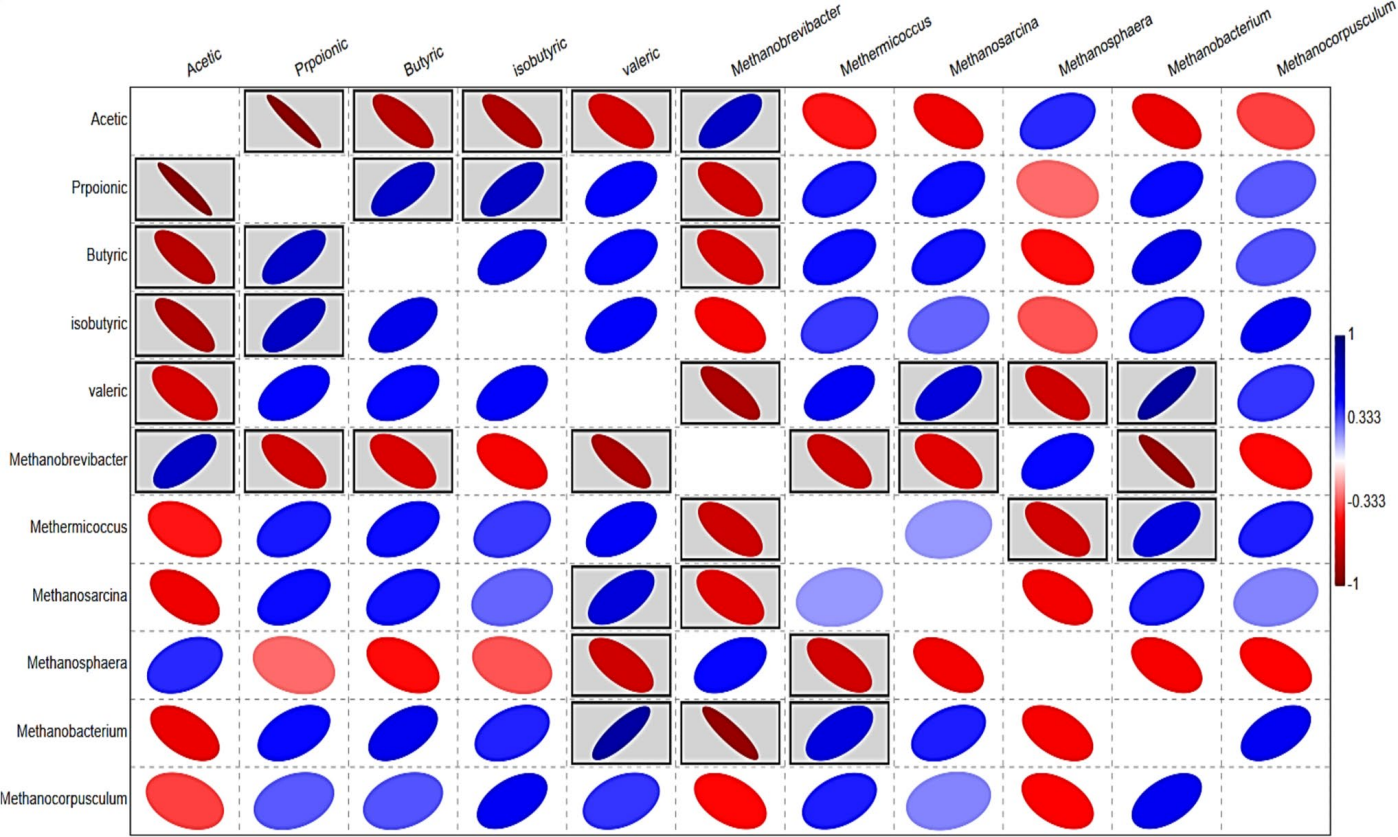
Heatmap based on Pearson’s correlation analysis between volatile fatty acids and rumen archaea. The black-boxed ellipses refer to the significant correlations at *P* < 0.05

Pearson correlation was conducted between relative abundances of rumen methanogens and concentration of VFAs. The correlation relationships were viewed as a heatmap (Fig. 5), which showed several negative and positive correlations. Genus *Methanobrevibacter* correlated positively with acetic acid and negatively with propionic acid. Also, valeric acid correlated negatively with *Methanobrevibacter* and positively with *Methanosarcina and Methanobacterium.*

## Discussion

### Rumen fermentation

Rumen microbiome, including rumen methanogens, is determined by animal diet and animal species. Methane production in the rumen impacts animal efficiency and increases global warming (Tseten et al. 2022). Therefore understanding the effect of different animal diets on the rumen methanogens in different animal species progresses the global efforts to cut methane emission and improve of livestock sector. Concentrate level impacts the fiber-to-starch ratio; consequently, it impacts the animal feed intake, volatile fatty acids, and the relative abundance of the dominant methanogenic genera, which is in the same line with the findings of previous studies on feedlot bulls and sheep (Popova et al. 2011; Rabee et al. 2022). However, no available data on the effect of concentrate level on the methanogenic community in dromedary camels. Alfalfa hay was used as a sole roughage source and offered to the animal on the ad-lib basis. Feed intake of the camels was in the range of camels reported by Laameche et al. (2019) and Khattab et al. (2021). Concentrate level did not impact the total VFA production or methanogens population which was also reported by Popova et al. (2011). The proportions of VFA showed an opposite trend and the values were similar to the values observed by Khattab et al. (2021). The increase in the dietary concentrate level increased the percentage of propionic acid and declined the acetic acid; a similar trend was observed in cows fed normal and low-roughage diets (Granja-Salcedo et al. 2016). Wang et al. (2018) indicated that increasing the forage-to-concentrate ratio increases the fiber content, which encourages the fibrolytic bacteria and increases acetic acid production. Rabee et al. (2020) reported that starchy diets encourage the amylolytic bacteria that produce propionic acid, which supports our results. These findings also were confirmed by a previous study by Askar et al. (2023) who investigated the effect of concentrate level on the bacterial community in camels and indicated that increasing the concentrate level, increased the relative abundance of the bacterial genus *Prevotella* that produces propionic acid. The same conclusion was reported by Wang et al. (2018) who studied the effect of roughage to concentrate ratio in cows.

### Structure of the community, diversity

Rumen methanogens are impacted by the rumen microbial fermentation of animal diet that provides the growth substrates for methanogens, which interact with the H_2_-producing and H_2_-utilizing microorganisms that are impacted by diet composition (Rabee et al. 2022). Alpha diversity indices (observed ASVs, Chao1, Shannon, Simpson, InvSimpson) and beta diversity (PCoA) of the methanogenic community were not influenced by diet type, which is similar to results on lambs, feedlot bulls, and dairy cows (Popova et al. 2011; Cersosimo et al. 2016; Rabee et al. 2022). However, the relative abundances of genus *Methanobrevibacter*, *Methanobacterium*, and *Methanosarcina* were impacted by increasing the concentrate level. Genus *Methanobrevibacter* dominated the methanogenic community in camels fed different concentrate levels, which is consistent with previous studies on different ruminant species, including dromedary camel (Seedorf et al. 2015; Rabee et al. 2020). This genus is the main methane producer in the rumen (Tapio et al. 2017) and was declined by increasing the concentrate levels. The variation of the relative abundances of rumen methanogens could be explained as a result of the availability of H_2_, CO_2_, and other growth substrates (acetic, butyric, formate, glucose….etc.) that are produced from the microbial fermentation of diet components (cellulose, hemicellulose, protein, pectin….etc.) (Jeyanathan et al. 2011), which explains the current findings and is supported by the results of PCA and explain the positive correlation between *Methanobrevibacter* and acetic acid and the negative correlation between this genus with propionic acid. Acetic acid provides a methyl group that methanogens use in methanogenesis; at the same time, the production of propionic acid in the rumen consumes the H_2_ molecules, which affect methanogenesis adversely (Tapio et al. 2017; Bharanidharan et al. 2018). *Methanosphaera* is one of the dominant methanogenic species in different animal species (Carberry et al. 2014b). The relative abundances of *Methanosphaera* and *Methanobrevibacter* were found higher in cattle with low methane emission (Smith et al. 2022). The decline in the relative abundance of *Methanobrevibacter* and *Methanosphaera* by decreasing the concentrate level was also reported by Zhu et al. (2017) who stated that *Methanosphaera* is hydrogen-dependent methylotrophs; it uses an H_2_ molecule to reduce methanol to produce methane. Therefore, the decline in the relative abundance of *Methanobrevibacter* and *Methanosphaera* could be explained by the depletion of H_2_, which is needed also in propionate production. Genus *Methanomassiliicoccus* has a great contribution to overall CH_4_ production; this genus was not detected in the C3 group, which is a positive point for higher concentrate levels (Pitta et al. 2022).

*Methanosarcina* uses a wide range of substrates to produce methane through hydrogenotrophic, acetoclastic, or methylotrophic pathways, which could justify the increase in the relative abundance by increasing the concentrate level (Lambie et al. 2015). Bowen et al., (2020) reported relationships between the relative abundances of rumen methanogenic genera and feed efficiency in finishing steers. At the same time, Zhou et al. (2009) reported that the prevalence of genus *Methanosphaera* and *Methanobrevibacter* was greater in inefficient animals, which could support our speculation.

## Conclusion

The camel group fed medium level (1%) of concentrates (C2) showed the lowest feed intake. Increasing the concentrate level declined the relative abundances of major methanogenic genera in the rumen of dromedary camels and affected the volatile fatty acid profile. Alleviating the concentrate level in animals’ diets could be a way to improve feed efficiency and decline the methane emission from growing camels.

## Data Availability

The datasets generated and/or analyzed during the current study are available in SRA at https://www.ncbi.nlm.nih.gov/sra/PRJNA1008431
